# A Comparative Evaluation of Postoperative Sensitivity Between Cention-N and Resin-Modified Glass Ionomer Cement in Class V Cavity: An In Vivo Study

**DOI:** 10.7759/cureus.47801

**Published:** 2023-10-27

**Authors:** Rudra R Bharate, Aditya S Patel, Amit Reche, Rishika C Dhimole

**Affiliations:** 1 Conservative Dentistry and Endodontics, Sharad Pawar Dental College, Datta Meghe Institute of Higher Education and Research, Wardha, IND; 2 Public Health Dentistry, Sharad Pawar Dental College, Datta Meghe Institute of Higher Education and Research, Wardha, IND

**Keywords:** aesthetics, dentinal hypersensitivity, microleakage, restorative material, non carious cervical lesion

## Abstract

Aim

This research aimed to assess and compare the postoperative sensitivity in class V cavity when restored with Cention-N and resin-modified glass ionomer cement (RMGIC). This study used the Schiff scale to assess sensitivity in the class V cavity.

Materials and methods

This was an in vivo study performed in the Department of Conservative Dentistry and Endodontics, Sharad Pawar Dental College and Hospital, Sawangi Meghe, Maharashtra. Patients included in this study were randomly selected from regular outpatient departments diagnosed with class V cavities and referred for restorative treatment. These patients were segregated into Group A and Group B. Demographic information, detailed history of any medical condition, dental history, any allergy related to resins, and another group of drugs were recorded. The in vivo study involved 36 patients, 18 in each group, who presented at Sharad Pawar Dental College and Hospital. Inform consent was taken from all patients. The subjects' responses were evaluated using a Schiff analog scale to assess postoperative sensitivity to an air blast stimulus.

Results

The study included all 36 patients aged between 18 and 40 years. All 36 patients were segregated into two distinct groups. Two groups of 18 patients in each group were restored with Cention-N and RMGIC in groups A and B, respectively. It was determined that RMGIC exhibits a relatively higher incidence of postoperative sensitivity than Cention-N. It was determined that the difference was statistically relevant.

Conclusion

Considering the limitations of this study, it can be concluded that RMGIC shows more postoperative sensitivity than other groups on the first, second, and seventh days. Thus, it is concluded that Cention-N can be a superior alternative to RMGIC.

## Introduction

Restorative dentistry tends to treat defective tooth structures and simultaneously restore the function and aesthetics of the tooth. Materials used for restoration should be non-toxic, non-irritant to vital pulp, biocompatible, dimensionally stable, wear-resistant, durable, and aesthetics. The primary concern of patients during restorative treatment is aesthetics, due to which different materials were introduced for restorative treatment. Lesions like Class III, IV, and V cavities, non-carious cervical lesions (NCCLs), and fractures are some reasons for poor aesthetics. Class V cavity occurs on labial, buccal, and occasionally lingual surfaces of teeth [1]. These lesions are superficial and usually affect only one surface of the tooth. Cervical lesions impact the tooth's structural integrity, increase bacterial plaque accumulation, increase dentinal hypersensitivity, and lead to poor aesthetic appearance [2]. NCCLs are a challenging dental problem that needs special assistance. NCCLs are attrition, abrasion, abfraction, and erosion, which are also responsible for Class V cavities [3]. Various materials are used in the restoration of the Class V cavity. A successful restorative material must provide an adequate seal at the tooth surface-restoration interface to prevent microleakage. Postoperative sensitivity, pulpal discoloration, marginal discoloration, secondary caries, bacterial penetration, and pulpal inflammation can all be consequences of poor adaptation of restorative material [4]. The durable hermetic seal which prevents microleakage should be created with restorative material [5]. One of the primary challenges associated with restoring Class V cavity is to attain a complete marginal seal. Restoration failure may result in microleakage, polymerization shrinkage, and hence increased postoperative sensitivity [6].

Composite resin is the most commonly used material in dental practices due to its aesthetic appeal and strong bonding properties with enamel and dentin [7]. Polymerization shrinkage is a considerable concern when using composite resin to restore Class V cavities. This leads to clinical complications, including poor marginal seal, fractured restoration, bonding system solubility, and marginal leakage [8]. Another material used to restore Class V cavity is glass ionomer cement (GIC), acid-base cement. The formation of GIC arises from a chemical reaction of mild polymeric acids and powdered glass. This reaction leads to the formation of a final structure that includes filler particles in the shape of unreacted glass, thus enhancing the strength of the set cement [9]. GIC is an adhesive bioactive restorative material with therapeutic action. GICs have found consistent clinical utilization due to their advantageous properties, including their ability to adhere to dentin, bond effectively with tooth structures, exhibit bactericidal properties, and demonstrate biocompatibility [10]. The main advantageous action of GIC is it releases fluoride, which promotes remineralization of tooth structure [11]. GIC has been noted for its ability to deliver effective marginal sealing, reduce microleakage, and have good retention ability [12]. However, conventional GICs have certain clinical drawbacks, including extended setting times, potential dehydration during the initial setting stage, and a textured surface that may compromise mechanical resilience [13].

To overcome these disadvantages of conventional GIC, recently advanced material in generating GIC is resin-modified glass ionomer cement (RMGIC). This material has been created to enhance the material's physical qualities and may benefit patients [14]. Compared to traditional GIC, RMGIC offers several benefits, including prolonged working time, enhanced setting time, adequate marginal seal, fluoride release, superior aesthetics, and higher strength [15]. RMGIC fluoride releasing property binds fluoride to tooth structure without additional bonding agent [16]. Recently, a newer material, Cention-N, was introduced, a highly aesthetic direct filling restorative material for anterior and posterior restorations. This self-curing restorative material primarily contains urethane dimethacrylate (UDMA) in powder and liquid form. Cention-N is a recently advanced alkasite restorative material, as it utilizes alkaline filler and isofiller that effectively minimize polymerization shrinkage. Cention-N is a tooth-colored self-curing restorative material with an added light-curing option [17]. Cention-N is considered a novel filling material with less polymerization shrinkage and microleakage than other materials mentioned above, leading to less postoperative sensitivity.

This research aims to assess and compare the postoperative sensitivity in the Class V cavity when restored with Cention-N and RMGIC. This study uses the Schiff scale to assess sensitivity in the Class V cavity.

## Materials and methods

Approval of this research was done by the Institutional Ethics Committee of Datta Meghe Institute of Higher Education and Research ref no DMIHER (DU)/IEC/2023/720. This was an in vivo study performed in the Department of Conservative Dentistry and Endodontics, Sharad Pawar Dental College and Hospital, Sawangi Meghe, Maharashtra. Patients included in this study were randomly selected from regular outpatient flow diagnosed with Class V cavities and referred for restorative treatment. These patients were segregated into Group A and Group B. Demographic information, detailed history of any medical condition, dental history, any allergy related to resins, and another group of drugs were recorded. The in vivo study involved 36 patients, 18 in each group, who presented at Sharad Pawar Dental College and Hospital. Inform consent was taken from all patients. Before the treatment, patients with gingival inflammation were treated and then evaluated as part of the pre-operative assessment. During restoration, a strict isolation protocol using a rubber dam was followed to exclude sensitivity from adjacent teeth. The subjects' responses were evaluated using a Schiff analog scale to assess postoperative sensitivity to an air blast stimulus. This scale assigns scores ranging from 0 to 3 to participants. The scoring criteria for the Schiff analog scale are the following: Grade 0 if the subject does not respond to the air blast stimulus, Grade 1 if the subject responded to the air blast stimulus but did not request to discontinue the stimulation, Grade 2 if the subject responded to the air blast stimulus and asked to discontinue the stimulation or moved away from the stimulation, and Grade 3 is subject responded to air blast stimulus, considers it as painful and ask to discontinue the stimulation. Patients exhibiting pre-operative scores of 2 or 3 on the Schiff scale were also incorporated into this research.

Estimation of sample size

The sample size is calculated using the following formula [18]:

n= Z1-α/2+Z1-β) 2p1 (1-p1) +p2 (1 - p2) / (p1 - p2)2

Where Z1-α/2 is the confidence interval of 95%, confidence interval Z1 is 1.96, Ζβ is the critical value of normal distribution at β = 0.95, d= difference to be detected, 1.0-0.1 =0.9, σ, population standard deviation, SD1=0.37, SD2=0.48. The total sample size is 36. With the calculation mentioned above, the sample size determination of both the groups is 18 in the RMGIC group and 18 in the Cention-N group, a total minimum sample size with a 95% confidence interval. The total sample size was determined to be 36, evenly distributed as 18 participants in the RMGIC group and 18 in the Cention-N group. The first 18 patients in this study were included in the Cention-N group, and the second 18 were registered in the RMGIC group.

Inclusion criteria

Only patients who voluntarily opted for restorative procedures were included in the research study and provided adequate time to undergo the procedure. The study included individuals aged 18 to 40 years, encompassing all cases of maxillary and mandibular teeth presenting Class V cavities with cavity depths ranging from 1 to 3 mm. Furthermore, patients exhibiting pre-operative scores of 2 or 3 on the Schiff scale were also incorporated into this research. Patients with gingival inflammation were treated, and the gingiva was inflammation-free before treatment of the cavity.

Exclusion criteria

The study excluded patients who were periodontally compromised, had any pulpal disease and periapical pathology, and consumed analgesics and anti-inflammatory medications. Patients with known allergies to the resin material and other substances were excluded from the study. Those exhibiting undesirable habits like bruxism and clenching were also excluded.

Intervention

Class V tooth preparations are situated in the gingival one-third of the tooth in the region of the Class V cavity. All 36 patients were segregated into two distinct groups. Two groups of 18 patients in each group were restored with Cention-N and RMGIC in groups A and B, respectively. Isolation from saliva, adjacent teeth, and other intraoral fluids was done to exclude sensitivity from adjacent teeth using a rubber dam. The cavity was prepared using a round bur. Class V cavities with a cavity depth of 1 to 3 mm were prepared during cavity preparation. A periodontal probe measured cavity depth. The three-way syringe was used to clean the lesion. Manipulation of the materials present in powder and liquid form was performed. The material was restored in the cavity using a cement carrier. Removal of excessive material using a carver was performed. After the procedure, patients received postoperative guidelines, which included instructions on maintaining oral hygiene. Follow-up appointments were scheduled for patients on the first, second, and seventh days after the procedure to assess postoperative dentin hypersensitivity. The sensitive tooth was isolated during evaluation, and an air blast, administered with a three-way syringe at approximately 40 psi, was directed at the restored tooth surface. The patient's reaction to this air blast stimulus was assessed using the Schiff analog scale mentioned earlier.

## Results

This study includes all 36 patients, subsequently divided into groups A and B, each consisting of 18 patients. Table 1 represents the demographic characteristics of both groups. Statistical analysis revealed no significant association between age (p=0.3529) and gender (p=0.3166).

**Table 1:** Statistical analysis for demographic details in two groups

Demographic details	Group A	Group B	Chi^2 ^test	p-value
Age group (18-40)				
18-25	5	3		
26-33	4	8	2.0833	0.3529
34-40	9	7		
Gender (male/female)				
Male	8	11	1.0031	0.3166
Female	10	7		

Table 2 represents pre-operative clinical findings for groups A and B, including parameters like the cavity depth, pre-operative sensitivity score, and the extent of gingival inflammation. Statistical analysis showed non-significant associations for depth of cavity (p=0.5023), pre-operative sensitivity score (p=0.3166), and gingival inflammation (p=0.2888).

**Table 2:** Statistical analysis of pre-operative clinical findings in two groups

Pre-operative clinical findings	Group A	Group B	Chi^2 ^test	p-value
Depth of Class V cavity				
1) 1-2 mm	11	9	0.45	0.5023
2) 3 mm	7	9		
Pre-operative sensitivity score (using Schiff scale)				
1) Score 2	11	8	1.0031	0.3166
2) Score 3	7	10		
Gingival inflammation				
1) Patients with no gingival inflammation	15	17	1.125	0.2888
2) Patients treated with gingival inflammation before restoration	3	1		

Two groups of 18 patients in each group were restored with Cention-N and RMGIC in groups A and B, respectively. Pre-operative dentinal sensitivity was assessed using a Schiff scale on patients in both groups. The data presented in Table 3 is an association of pre-operative sensitivity among groups A and B using an unpaired T-test. Table 3 concludes that pre-operative sensitivity among Group A (2.5556±0.51131) was slightly higher than that of Group B (2.5000±0.51450). This association was found to be statistically non-significant (p=0.747).

**Table 3:** Statistical analysis of pre-operative sensitivity in Class V cavity N: number, %: percentage

Variables	N	Mean	Std. deviation	Std. error mean	Std. error difference	95% confidence interval of the difference		t-value	p-value
						Lower	Upper		
Group A	18	2.5556	0.51131	0.12052	0.17097	0.29189	0.40301	0.325	0.747
Group B	18	2.5000	0.51450	0.12127	0.17097	0.29189	0.40301		

Postoperative dentinal sensitivity in the Class V cavity was assessed using a Schiff scale on the first, second, and seventh days. The data presented in Table 4 is an association of Cention-N and RMGIC cement using an unpaired T-test. Table 4 shows that on day 1, the sensitivity of RMGIC (2.2222±0.64676) was higher than that of Cention-N (1.5556±0.70479); this comparison was found to be statistically significant for day one (p=0.006). On day 2, the sensitivity of RMGIC (1.7222±0.82644) was higher than that of Cention-N (0.8889±0.67640). This comparison was statistically significant for day two (p=0.002). On day 7, the sensitivity of RMGIC (1.3889±1.14475) was higher than that of Cention-N (1.0000±1.14475). This comparison was statistically significant for day seven (p=0.004).

**Table 4:** Statistical analysis of postoperative sensitivity in Class V cavity on days 1, 2, and 7 when restored with Cention-N and RMGIC N: number, %: percentage, RMGIC: resin-modified glass ionomer cement

Variables		N	Mean	Std. deviation	Std. error mean	95% confidence interval of the difference		t-value	p-value
						Lower	Upper		
Day 1	Cention-N	18	1.5556	0.70479	0.16612	1.12487	0.20846	3.311	0.006
	RMGIC	18	2.2222	0.64676	0.15244	1.12499	0.20834		
Day 2	Cention-N	18	0.8889	0.67640	0.15943	1.34489	0.32178	2.957	0.002
	RMGIC	18	1.7222	0.82644	0.19479	1.34563	0.32104		
Day 7	Cention-N	18	1.0000	0.97014	0.22866	1.10766	0.32988	1.100	0.004
	RMGIC	18	1.3889	1.14475	0.26982	1.10837	0.33059		

From Table 5, we conclude that RMGIC had comparatively higher sensitivity incidences than Cention-N when used as a restorative material in Class V cavity.

**Table 5:** Comparison between postoperative sensitivity in Class V cavity when restored with Cention-N and RMGIC N: number, %: percentage, RMGIC: resin-modified glass ionomer cement

Variables	N	Mean	Std. deviation	Std. error mean		95% confidence interval of the difference		t-value	p-value
						Lower	Upper		
Pre-operative sensitivity in Group A patients	18	2.5000	0.51450	0.12127	0.29582	0.50993	1.71229	3.756	0.001
Postoperative sensitivity in Group A patients restored with RMGIC	18	1.3889	1.14475	0.26982	0.29582	0.50002	1.72220		
Pre-operative sensitivity in Group B patients	18	2.5556	0.51131	0.12052	0.25848	1.03026	2.08085	6.018	0.000
Postoperative sensitivity in Group B patients restored with Cention-N	18	1.0000	0.97014	0.22866	0.25848	1.02401	2.08710		

## Discussion

Dentists frequently encounter NCCLs, which present considerable challenges in dentistry. Selecting appropriate restorative materials is key to a successful restoration [19]. As adhesive dentistry continues to evolve with innovative techniques and principles, there is a growing preference for esthetic restorative material, particularly in cases where aesthetic considerations are paramount. Despite recent advancements in dental adhesives and composite resin materials, several persistent challenges remain. These challenges include microleakage, postoperative dentinal sensitivity, and the risk of secondary caries. These issues pose a risk of eventual failure in restoration [20]. GIC is still regarded as the only material known for its self-adhesive properties in dental tissues. Recently, there have been continuous advancements in developing new formulations of GIC to address certain clinical limitations, focusing on enhancing its physical properties [5]. Dentists have been searching for a viable alternative to GIC for quite some time. This option is cost-effective, can release fluoride, and is quick and straightforward to use without complex equipment. Cention-N, a recently introduced restorative material, meets these criteria and offers several advantages over traditional composite and GIC. Cention-N boasts superior strength compared to GIC. This dual-cured, tooth-colored material is well-suited for deciduous and permanent dentition restoration, encompassing all cavities. Its versatility and ease of use are evident as it can be utilized both with and without an adhesive.

In a research performed by Meshram et al., they investigated microleakage in a Class V cavity filled by Cention-N and flowable composite resin. The study showed microleakage, but the differences seen between these two groups were not statistically significant. The group that used Cention-N in combination with an adhesive exhibited the lowest level of microleakage than the group using flowable composite. Conversely, it was observed that Cention-N without the adhesive showed a higher level of microleakage [21]. The study by Shenoi et al. compared microleakage in Class V cavity in which restoration was done using a composite of flowable consistency and Cention-N. In summary, Cention-N shows substantially less microleakage and improved adaptability than flowable composite, as the statistical analysis supports [22].

Research conducted by Sujith et al. aimed to differentiate the physical properties and microleakage of Cention-N compared with GIC and hybrid composite restorative materials. The study concluded that Cention-N showed less flexural strength than GIC [23]. A study conducted by Mushtaq carried out a comparative evaluation to assess postoperative sensitivity after restoring Class I lesions using different restorative materials. The study findings revealed that postoperative sensitivity ranked the lowest in GC Fuji IX and highest in nano-hybrid composite with the help of etch-and-rinse adhesive [24]. The main limitation of this research was it was conducted on the Class I cavity, whereas sensitivity is most commonly encountered in the Class V cavity.

The current research evaluated the postoperative sensitivity of Cention-N and RMGIC in Class V cavities. The study showed that Cention-N exhibited lower postoperative sensitivity compared to RMGIC. Consistent with multiple studies, Cention-N demonstrated reduced microleakage properties compared to RMGIC, which likely contributed to its lower incidence of postoperative dentinal sensitivity. Additionally, Cention-N has the advantage of shorter chair-side time compared to RMGIC.

Limitations

This study had a sample size of just 36 patients, exclusively focusing on Class V cavities and employing a single filling technique (incremental). Furthermore, the follow-up period was limited to just seven days.

## Conclusions

Research on a comparative evaluation of postoperative sensitivity between Cention-N and RMGIC in Class V cavity restoration, an in vivo study, was conducted on 36 patients. Considering the limitations of this study, it can be concluded that RMGIC shows more postoperative sensitivity than Cention-N on the first, second, and seventh days. Observational findings suggested that Cention-N shows a superior marginal seal than RMGIC, preventing microleakage and reducing postoperative sensitivity.
